# College and Hospital Notes

**Published:** 1908-11

**Authors:** 


### COLLEGE AND HOSPITAL NOTES

Changes in the staff of the Buffalo General Hospital are announced as follows: Dr. John Parmenter, from attending surgeon to consulting surgeon; Dr. Edgar R. McGuire, from assistant attending surgeon to associate surgeon. Dr. Frederick J. Parmenter was appointed assistant surgeon.

Changes in the staff at the German Deaconess's Hospital recently have been numerous for some unexplained reason. New members of the medical staff suddenly received notice from the management that their services were at an end. Among those who are dropped occur the names of the following well-known physicians: Dr. Conrad Diehl, Dr. DeLancey Rochester, Dr. James W. Putnam, Dr. William Gaertner, Dr. W. S. Renner and Dr. Edmond E. Blaauw.

The new appointees are as follows: Dr. David A. Morrison, Dr. Henry E. Stadlinger, Dr. Nelson W. Bodenbender, Dr. George L. Fischer, Dr. M. J. Downey, Dr. Alice A. Bennett, Dr. George W. Seitz, Dr. Max Breuer and Dr. Leonard Reu.

Japanese progressiveness is again emphasised by the fact that in October 25, 1908, a magnificent hospital built by the Japanese Government, was formally opened at Seoul, Corea. It is one of the finest institutions in the Far East and includes a school of medicine.

The new Homeopathic Hospital, to be erected at the corner of Linwood and Lafayette avenues, Buffalo, has become an assured fact. Mrs. George A. Plimpton, president of the advisory board, dug and turned the first spadeful of earth on Tuesday morning, October 20, 1908, whereupon the contractor immediately began active work, with the expectation that the building will be ready for occupancy in the spring of 1910. The ceremony was witnessed by a number of citizens, besides the officials who were present.

When Mrs. Plimpton had turned the sod with the aid of a pick, her daintily decorated spade being too light for the task, she expressed the hope that those who have been skeptical about the success of the hospital will now believe it is a reality, and put their shoulders to the wheel to help the good work along.

